# Oferta de servicios de atención farmacéutica: clave para un nuevo modelo de servicios de salud

**DOI:** 10.1016/j.aprim.2021.102198

**Published:** 2021-10-21

**Authors:** Begoña Calvo Hernáez, Miguel Ángel Gastelurrutia Garralda, Amaia Urionagüena de la Iglesia, Arantxazu Isla Ruiz, Ana del Pozo Rodríguez, María Ángeles Solinís Aspiazu

**Affiliations:** a Pharmacy Practice Research Group, Faculty of Pharmacy, University of the Basque Country, UPV/EHU, Vitoria-Gasteiz, Spain; b Bioaraba, Pharmacokinetic, Nanotechnology and Gene Therapy Group, Vitoria-Gasteiz, Vitoria-Gasteiz, Spain

**Keywords:** Atención farmacéutica, Servicios farmacéuticos asistenciales, Farmacéuticos, Farmacia comunitaria, Colaboración interprofesional, Atención primaria de salud, Pharmaceutical care, Pharmaceutical care services, Pharmacists, Community pharmacy, Interprofessional collaboration, Primary health care

## Abstract

La colaboración interdisciplinar entre profesionales sanitarios es esencial para mejorar los resultados en salud de la población. Las capacidades que poseen los farmacéuticos les convierten en profesionales que pueden contribuir al control integral de la farmacoterapia en coordinación con otros profesionales de la salud. El Consejo de Europa aprobó la resolución CM/Res(2020)3 sobre implementación de la atención farmacéutica en el sistema sanitario para promover el uso apropiado y seguro de los medicamentos. Entre las actividades a realizar mediante el proceso de atención farmacéutica destaca la detección de problemas relacionados con la farmacoterapia como son las contraindicaciones, duplicidades, errores de prescripción, interacciones, etc. La intervención farmacéutica en ese ámbito requiere establecer un marco de colaboración interprofesional adecuado. En el presente artículo se comentan los aspectos a abordar para afrontar el cambio de modelo hacia una farmacia asistencial, con una mayor integración e implicación en el sistema, todo ello bajo el amparo del Consejo de Europa.

## Abstract (en)

Interdisciplinary collaboration between health professionals is essential to improve health outcomes. The competences of pharmacists make them professionals capable of contributing to the comprehensive management of pharmacotherapy in collaboration with other healthcare professionals. The Council of Europe adopted resolution CM/Res(2020)3 on implementation of pharmaceutical care for the benefit of patients and health services in order to promote the appropriate and safe use of medicines. One of the activities to be carried out through the pharmaceutical care process is the detection of drug related problems, such as contraindications, duplications, prescription errors, interactions, etc. In many cases, it involves regular patient follow-up and needs an evaluation of the interventions performed, requiring the establishment of an appropriate interprofessional collaboration framework. This article discusses the issues to be addressed to face the change of model towards a care-based pharmacy.

## Integración de la atención farmacéutica en el sistema sanitario

Actualmente, la prevalencia de enfermedades crónicas en nuestra sociedad va en aumento, de forma paralela al envejecimiento de la población, lo que supone un reto de adaptación para los sistemas sanitarios. Por otra parte, a pesar de la existencia de tratamientos farmacológicos eficaces para la mayoría de las patologías crónicas, en muchos casos no existe un buen control de la enfermedad, lo cual conlleva la utilización de recursos sanitarios adicionales y gastos evitables. En este sentido, entre las principales causas evitables se encuentran los errores de medicación o la falta de adherencia al tratamiento^1^, ^2^, ^3^.

Las capacidades y competencias que poseen los farmacéuticos les convierten en profesionales que pueden contribuir de forma efectiva al control integral del tratamiento farmacoterapéutico en coordinación con los médicos, enfermeros y farmacéuticos de los equipos de Atención Primaria, tal como se recoge en el Marco Estratégico para la Atención Primaria y Comunitaria^4^. Una de las grandes ventajas del modelo de farmacia en nuestro país es la accesibilidad, ya que gracias a su distribución territorial la farmacia se constituye en una red que puede realizar servicios de atención farmacéutica junto a programas de salud comunitaria, incluidas las campañas de promoción de la salud y diferentes cribados.

El Consejo de Europa aprobó en 2020 la resolución CM/Res(2020)3 sobre la implementación de la atención farmacéutica en el sistema sanitario con el fin de beneficiar a los pacientes y a los servicios de salud^5^. Según la definición de Hepler et al., la atención farmacéutica consiste en la provisión responsable de la farmacoterapia con el fin de lograr resultados que mejoren la calidad de vida de los pacientes^6^. Ello conlleva además de la colaboración profesional farmacéutico-paciente, la coordinación con el resto de los profesionales de la salud en el diseño, implementación y seguimiento de los planes terapéuticos para obtener resultados positivos en salud.

La resolución pone en evidencia el valor añadido de la atención farmacéutica basada en una atención centrada en el paciente y en el uso eficiente de los medicamentos y proporciona una base legal a las autoridades sanitarias y a profesionales para implementar los métodos de trabajo relacionados con este enfoque en la práctica diaria. Por otra parte, la resolución deja claro que las actividades de atención farmacéutica se deben realizar, además de las actividades y funciones que actualmente realizan los profesionales farmacéuticos, en la gestión del suministro de medicamentos, incluidos su dispensación y la garantía de calidad.

## Servicios farmacéuticos asistenciales

La resolución citada describe el proceso de la atención farmacéutica que supone la realización de las siguientes actividades**:**

1. Evaluación de las necesidades relacionadas con la medicación, mediante su revisión estructurada, para detectar si existe algún problema relacionado con la farmacoterapia.
  Esta evaluación se facilitaría si el farmacéutico pudiera acceder y contribuir a los registros pertinentes sobre la salud del paciente, contando siempre con la autorización expresa del mismo, mediante la firma de un consentimiento informado, y centrando la información fundamentalmente en los datos relacionados con sus necesidades farmacoterapéuticas: estado general de salud, resultados de análisis de laboratorio, enfermedades crónicas y adherencia a la medicación, entre otros. Mediante los diferentes servicios profesionales ofertados en la farmacia, pueden detectarse problemas relacionados con la medicación (PRM) que necesitarán ser comunicados al personal médico o a otros profesionales sanitarios. Entre esos PRM se encuentran contraindicaciones, dosis/pauta/duración no adecuadas, duplicidades, errores en la prescripción, interacciones, alta probabilidad de efectos adversos u otros problemas de salud que afectan al tratamiento, etc.
2. Seguimiento regular de pacientes para evaluar los resultados de las intervenciones realizadas, utilizando medios de comunicación adecuados que incluyen la consulta periódica en la farmacia. Los resultados de las consultas de seguimiento deben documentarse y comunicarse en caso necesario al resto de profesionales implicados.
3. Consejo farmacéutico, educación y asesoramiento a pacientes, para lograr el uso óptimo de la medicación. Ello mejorará la adherencia y la seguridad del tratamiento farmacológico y facilitará su autocuidado.

Estas actividades, en la práctica, se materializan mediante la prestación de diferentes servicios profesionales. Así, la resolución describe algunos que ya se realizan en diferentes países, como son: a) el servicio de tratamiento de síntomas menores (indicación farmacéutica), b) el de nuevos medicamentos (*new medicines service*) para mejorar la adherencia terapéutica ante inicios de tratamientos, c) apoyo en patologías complejas y en personas vulnerables, realizando, por ejemplo, revisiones de la medicación, d) control de la seguridad de medicamentos de alto riesgo, como son los de «seguimiento adicional», e) servicios de prevención de la enfermedad mediante la realización de cribados, f) la monitorización de pacientes con enfermedades crónicas abordando, por ejemplo, la adherencia en pacientes con hipertensión o diabetes y g) los servicios de información de medicamentos, algo eminentemente farmacéutico y esencial para el conjunto del sistema sanitario. Algunos de estos servicios ya se están realizando en países como Inglaterra, Dinamarca o los Estados Unidos^7^, ^8^.

En nuestro país, al margen de los servicios clínicos desarrollados por farmacéuticos de Atención Primaria y de Farmacia Hospitalaria, actualmente se ofrecen algunos servicios asistenciales en las farmacias comunitarias que son financiados a nivel autonómico, provincial o municipal, los cuales varían en función de las autonomías y abarcan desde el servicio de dispensación de metadona, cribado de cáncer colorrectal o test de VIH, hasta el tratamiento observado directamente (TOD) en el que el profesional farmacéutico vigila especialmente el adecuado cumplimiento del tratamiento^9^.

En España, el Foro de Atención Farmacéutica en Farmacia Comunitaria, organización que incluye instituciones y organizaciones profesionales del ámbito de la farmacia, ha definido y clasificado los servicios asistenciales que podrían permitir atender las propuestas de la resolución europea, los cuales se recogen en la tabla 1^10^. Entre ellos se encuentran el servicio de indicación farmacéutica para tratamiento de síntomas menores, el de conciliación de la medicación en las transiciones de nivel asistencial o el servicio de adherencia terapéutica, entre otros.

**Tabla 1:** Clasificación de los servicios profesionales farmacéuticos asistenciales de la Farmacia Comunitaria*

Servicios de Atención Farmacéutica	Servicios relacionados con la salud comunitaria
Dispensación	Promoción de la salud
Indicación farmacéutica en síntomas menores	Educación sanitaria
Conciliación de la medicación	Prevención de la enfermedad incluyendo cribados y detección de patologías ocultas
Adherencia terapéutica	Medición de parámetros clínicos
Revisión de botiquines	Asesoramiento nutricional
Formulación magistral	Programa de intercambio de jeringuillas
Revisión del uso de los medicamentos	Deshabituación tabáquica
Asesoramiento sobre medicamentos	
Seguimiento farmacoterapéutico	
Farmacovigilancia	

* Servicios consensuados en Foro de Atención Farmacéutica en Farmacia comunitaria.10 Pueden existir otros servicios farmacéuticos no incluidos en la tabla.

## Impacto en la atención sanitaria

Existen revisiones sistemáticas de la literatura y metaanálisis que evalúan el impacto de los servicios de farmacia en la atención sanitaria primaria utilizando el modelo *Economic, Clinical and Humanistic Outcomes* (ECHO)^11^ para la evaluación de resultados en salud, los cuales han demostrado los efectos positivos de los servicios de farmacia tanto en los resultados para los pacientes como en los beneficios económicos para el sistema de salud^12^, ^13^. El servicio más estudiado ha sido el de revisión de la medicación con seguimiento farmacoterapéutico utilizando el método Dáder, metodología muy utilizada en la farmacia española para la realización de dicho servicio^14^, ^15^. El Consejo General de Colegios Oficiales de Farmacéuticos, también ha estudiado el impacto y la implementación de este servicio en el programa Consigue^16^, y de otros servicios como el de adherencia terapéutica en la Farmacia Comunitaria^17^. Por otra parte, la sociedad SEFAC está promoviendo la investigación en la farmacia comunitaria, habiendo liderado el proyecto internacional INDICA+PRO, codiseñado con médicos de Atención Primaria para evaluar el impacto y la implantación de un servicio de indicación en síntomas menores^18^. Todo ello apoya la aplicación en el ámbito de España de las recomendaciones contenidas en la resolución europea.

### Recomendaciones a los agentes implicados para su puesta en marcha

Sin embargo, para llevar a cabo la implantación de la atención farmacéutica y su posterior normalización^19^ y consiguiente desarrollo en el mundo real, la profesión farmacéutica debe entender la necesidad de centrar su actividad en la asistencia a los pacientes que utilizan medicamentos y no solo en el producto. La implantación de los servicios de atención farmacéutica requiere invertir tiempo del profesional farmacéutico y conlleva una mayor responsabilidad. Actualmente, en el sector de la farmacia comunitaria, la remuneración permanece vinculada al volumen de recetas dispensadas y/o al valor de los productos dispensados. Sin embargo, en los últimos años se ha reconocido que sería más adecuado remunerar la farmacia en base a la prestación de estos servicios^20^. Ello requiere reunir la evidencia de la contribución de los farmacéuticos comunitarios en la atención centrada en el o la paciente. En este sentido, existen estudios que han demostrado una relación favorable coste-efectividad y coste-beneficio de diferentes servicios asistenciales farmacéuticos^18^, ^21^, ^22^.

En segundo lugar, la farmacia comunitaria debe avanzar hacia su integración en el sistema de salud mejorando la comunicación interprofesional (para aumentar el conocimiento mutuo, y por tanto el respeto y la confianza), y pasar después a establecer un marco de colaboración interprofesional que cumpla determinadas condiciones. Por ello, debería abordarse un cambio de cultura en la práctica farmacéutica, así como cambios formativos e, incluso, legislativos.

Ese marco de colaboración debe favorecer el desarrollo de las necesarias relaciones interprofesionales. Se deben elaborar protocolos conjuntos en los que se definan claramente los roles de cada participante en base a sus competencias profesionales y las tareas a desarrollar por cada uno de ellos. Asimismo, es fundamental conocer y respetar los límites de actuación de los distintos profesionales implicados en el proceso. En este sentido, actualmente se están definiendo las competencias profesionales de los farmacéuticos, las cuales, junto a las competencias académicas definidas en la orden CIN correspondiente^23^, servirán de apoyo para la práctica de los servicios asistenciales.

Todos los agentes que participan en el proceso de uso de medicamentos deberían compartir la información necesaria relativa al paciente. Para ello, actualmente disponemos de herramientas que pueden facilitar la prestación de atención farmacéutica como son la receta electrónica y la historia clínica única y compartida, además de las tecnologías actuales de comunicación.

Los protocolos de colaboración entre los equipos de Atención Primaria y los farmacéuticos comunitarios, deberían beneficiarse de la coordinación del personal farmacéutico de Atención Primaria y permitirían obtener resultados en salud en actividades como la optimización de la farmacoterapia, la conciliación de la medicación, el aumento de la seguridad y efectividad de los tratamientos, la mejora de la adherencia y la reducción de la utilización inadecuada de los medicamentos. Para todo ello, la formación clínica del profesional farmacéutico comunitario debe actualizarse. Por tanto, y con el fin de asegurar que los farmacéuticos adquieran los conocimientos y las habilidades para llevar a cabo los servicios de atención farmacéutica deberían planificarse programas de formación en posgrado para el desarrollo profesional que permitan la recertificación continua del farmacéutico/a. Las facultades de farmacia deben estar bien alineadas con el resto de las facultades de ciencias de la salud y se debe potenciar, como ya se ha dicho, tanto la formación clínica como en atención farmacéutica.

Por todo ello, la farmacia asistencial, independientemente de su entorno, debe encontrar cabida como pieza clave para la gestión y asistencia sanitaria donde la provisión de servicios profesionales farmacéuticos, así como el apoyo de la farmacia comunitaria al modelo asistencial público y al uso racional del medicamento, proporcionen beneficios tanto clínicos como económicos y humanísticos.

En este sentido, para posibilitar una implantación real de las recomendaciones incluidas en la resolución, se deben desarrollar en el ámbito regional y nacional normativas para integrar la atención farmacéutica en los servicios de salud. La adopción de una nueva práctica profesional farmacéutica requiere del impulso y apoyo de la administración y de los agentes sanitarios. Sería conveniente redefinir dentro del Sistema Nacional de Salud la prestación farmacéutica que al día de hoy consiste básicamente en la dispensación de medicamentos, de forma que también se incluyan en la misma servicios asistenciales remunerados. Ello se facilitaría mediante el acceso a la historia clínica única y compartida.

## Conclusión

La publicación de la resolución del Consejo de Europa CM/Res(2020)3 debería servir para concienciar a los distintos actores implicados en el impulso de la atención farmacéutica. Desde la profesión farmacéutica, y desde los colegios oficiales de farmacéuticos, se deberá afrontar el reto que supone este cambio de modelo hacia una farmacia eminentemente «asistencial» en coordinación con los organismos sanitarios involucrados. Ello permitiría aprovechar el gran potencial que poseen los profesionales farmacéuticos para contribuir a la atención integral de las y los pacientes y a una mejor sostenibilidad del Sistema Nacional de Salud^24^.

Nos encontramos ante una gran oportunidad para impulsar el cambio en la gestión y asistencia sanitaria, con una mayor integración e implicación de las y los farmacéuticos en el sistema, todo ello bajo el amparo del Consejo de Europa.

## Financiación

Este trabajo ha sido financiado por la Universidad del País Vasco UPV/EHU (GIU 17/32) y proyecto Universidad-Sociedad US20/08.

## Conflicto de intereses

Los autores declaran no tener ningún conflicto de intereses.
